# Prognostic Values of Serum Ferritin and D-Dimer Trajectory in Patients with COVID-19

**DOI:** 10.3390/v13030419

**Published:** 2021-03-05

**Authors:** Fares Qeadan, Benjamin Tingey, Lily Y. Gu, Ashley Hafen Packard, Esther Erdei, Ali I. Saeed

**Affiliations:** 1 Department of Family and Preventive Medicine, School of Medicine, University of Utah, Salt Lake City, UT 84108, USA; Benjamin.Tingey@utah.edu (B.T.); lily.gu@utah.edu (L.Y.G.); Ashley.Hafen@utah.edu (A.H.P.); 2 Community Environmental Health Program, College of Pharmacy, Health Sciences Center, University of New Mexico, Albuquerque, NM 87106, USA; EErdei@salud.unm.edu; 3 Norton Thoracic Institute, St. Joseph’s Hospital and Medical Center, Phoenix, AZ 85013, USA; Ali.Saeed@DignityHealth.org

**Keywords:** COVID-19, cytokine storm, ferritin, D-dimer, trajectory, optimal prognostic cutoffs

## Abstract

Cytokine storm syndrome in patients with COVID-19 is mediated by pro-inflammatory cytokines resulting in acute lung injury and multiorgan failure. Elevation in serum ferritin and D-dimer is observed in COVID-19 patients. To determine prognostic values of optimal serum cutoff with trajectory plots for both serum ferritin and D-dimer in COVID-19 patients with invasive ventilator dependence and in-hospital mortality. We used retrospective longitudinal data from the Cerner COVID-19 de-identified cohort. COVID-19 infected patients with valid repeated values of serum ferritin and D-dimer during hospitalization were used in mixed-effects logistic-regression models. Among 52,411 patients, 28.5% (14,958) had valid serum ferritin and 28.6% (15,005) D-dimer laboratory results. Optimal cutoffs of ferritin (714 ng/mL) and D-dimer (2.1 mg/L) revealed AUCs ≥ 0.99 for in-hospital mortality. Optimal cutoffs for ferritin (502 ng/mL) and D-dimer (2.0 mg/L) revealed AUCs ≥ 0.99 for invasive ventilator dependence. Optimal cutoffs for in-house mortality, among females, were lower in serum ferritin (433 ng/mL) and D-dimer (1.9 mg/L) compared to males (740 ng/mL and 2.5 mg/L, respectively). Optimal cutoffs for invasive ventilator dependence, among females, were lower in ferritin (270 ng/mL) and D-dimer (1.3 mg/L) compared to males (860 ng/mL and 2.3 mg/L, respectively). Optimal prognostic cutoffs for serum ferritin and D-dimer require considering the entire trajectory of laboratory values during the disease course. Females have an overall lower optimal cutoff for both serum ferritin and D-dimer. The presented research allows health professionals to predict clinical outcomes and appropriate allocation of resources during the COVID-19 pandemic, especially early recognition of COVID-19 patients needing higher levels of care.

## 1. Introduction

The novel coronavirus (SARS-CoV-2) is associated with high morbidity and mortality, especially among those with underlying health conditions. COVID-19 is an infectious disease that is caused by a novel enveloped RNA virus, resulting in severe pneumonia [1]. Elevated serum ferritin and D-dimer levels are found in patients with cytokine storm syndrome (CSS) due to COVID-19 infection [2,3]. This article seeks to identify optimal serum cutoff levels for ferritin and D-dimer with trajectory plots of repeated values obtained during hospitalization as biomarkers for COVID-19 related invasive ventilator dependence (IVD) and in-hospital mortality. No optimal serum biomarker exists for COVID-19 infection.

Serum ferritin is an iron storage protein with the primary role of regulating cellular oxygen metabolism. Ferritin is composed of two different subunits, H and L. Previous studies have suggested H-ferritin acts as an immune modulatory molecule with both proinflammatory and immunosuppressive functions [4]. Increased ferritin levels could be indicative of a strong inflammatory reaction related to viral entry into the human body and its impact on iron metabolism [4]. According to Eloseily et al., an elevated ferritin value (e.g., >700 ng/mL) should alert clinicians to additional diagnostic work-up so that therapeutic approaches can be considered without significant delay. Early institution of treatment for CSS has been proven to lead to better patient outcomes [5]. Elevated ferritin serum levels have been found to significantly correlate with disease severity in COVID-19 infected patients [6]. A prior study found that, compared to moderate cases of COVID-19, severe cases had higher ferritin levels [7]. 

Several recent studies have also found associations between D-dimer levels and the severity of COVID-19 infection and mortality [2]. D-dimer is a degradation product of cross-linked fibrin, indicating increased thrombin generation and fibrin dissolution by plasmin. High D-dimer levels are common in acutely ill individuals with a number of infectious and inflammatory diseases [8]. Studies have indicated that the coagulation system is active in critically ill patients, and D-dimer levels correlate with activation of the proinflammatory cytokine cascade leading to CSS [9]. It is believed that coagulation abnormalities related to elevated levels of D-dimer cause venous thromboembolism, which may contribute to respiratory deterioration related to COVID-19 infection [10]. 

Most of these studies show significant elevation in serum ferritin and D-dimer in COVID-19 infected patients, yet they fail to identify an optimal serum cutoff level that can reliably predict the development of CSS resulting in acute lung injury with IVD and in-hospital mortality. Optimal serum cutoff level for ferritin and D-dimer along with trajectory plots will help with allocation of hospital resources based on individual clinical course.

## 2. Materials and Methods

### 2.1. Settings

We used data from the Cerner COVID-19 de-identified data cohort, which is a subset of the entire Cerner Real-World Data cohort. *“Cerner Real-World Data is extracted from the EMR of hospitals in which Cerner has a data use agreement. Encounters may include pharmacy, clinical and microbiology laboratory, admission, and billing information from affiliated patient care locations. All admissions, medication orders and dispensing, laboratory orders and specimens are date and time stamped, providing a temporal relationship between treatment patters and clinical information. Cerner Corporation has established Health Insurance Portability and Accountability Act-compliant operating policies to establish deidentification for Cerner Real-World Data”* [11]. 

Only patients with a confirmed COVID-19 diagnosis or recent positive lab test from January through June 2020 and valid ferritin or fibrin D-dimer laboratory results were included. These values were identified by Logical Observation Identifiers Names and Codes (LOINC) codes (Supplemental Table S1) and were considered valid if they were associated with encounters showing a diagnosis or recent positive lab test of COVID-19. Additionally, an independent dataset from July to September 2020 was used for validation analyses. The University of Utah Institutional Review Board (IRB #136696) has determined that this study does not meet the definitions of Human Subjects Research for using secondary data with no intervention or interaction with an individual, and for not having identifiable private information in the data.

### 2.2. Measurements

The outcomes of interest involved two different indications of clinical complications in COVID-19 patients: IVD, and in-hospital mortality. IVD was a binary indication (yes or no) representing whether a patient ever had a diagnosis, procedure, or encounter result that signified reliance on an invasive ventilator. The list of codes is found in Supplemental Table S2. These were kept separate from indications of less severe ventilator dependence such as continuous positive airway pressure (CPAP) and bilevel positive airway pressure (BiPAP) machines. In-hospital mortality was a binary indication (yes or no) of whether a patient died at hospital discharge.

The predictors of interest were ferritin and fibrin D-dimer lab results. These values were continuous, and all ferritin results were converted to be on the same unit-of-measure scale of nanograms per milliliter (ng/mL). All D-dimer results were converted to be on the same scale of milligrams, fibrinogen equivalent units, per liters (mg {FEU}/L). Measurements were sorted by date to provide a trajectory of results across the patient’s hospital stay.

Other predictors included gender and an indication of comorbidity based on the weighted Charlson comorbidity index (CCI) [12]. The CCI measures patient comorbidity by calculating a risk-assessment score based on ICD-10 diagnosis codes (Supplemental Table S3) that are associated with 17 chronic diseases. These specific diseases are listed in Table 1 [13]. With access to disease histories of patients as far back as January 1, 2015, the CCI considered all registered disease codes that fell in this time frame, using which to provide an index by categorizing numerical scores into the following categories: 0, 1–2, 3–4, and ≥5 [14]. The level of “0” is indicative of no disease burden, whereas “≥5” is indicative of maximal disease burden. Other demographic and clinical characteristics were included for descriptive analysis, including a continuous predictor (age in years), and categorical predictors (gender, race/ethnicity, insurance, and zip-code region). 

### 2.3. Statistical Analysis

Categorical variables were presented with frequencies and percentages, while continuous variables were presented with medians and interquartile ranges (IQR = Q1–Q3). Primary analyses were conducted separately for the ferritin and D-dimer cohorts. In each cohort, mixed-effects logistic regression models were fit with the respective laboratory values (ferritin or D-dimer) as a predictor, and the patient identifier as a random effect to allow modeling the trajectory of laboratory results within patients. For each patient, we have considered their trajectory of values instead of a single measurement, such as most recent or maximal measurement, to improve models’ predictability power. Lab values were transformed to the log-scale for violating normality. Results were presented back on their original scale by exponentiation. 

Assuming each patient as a cluster with correlated observations, we used logistic random-effects models [15] to calculate sensitivities and specificities, which in turn helped us identify the optimal cutoff level of the continuous predictor (ferritin or D-dimer lab) that discriminated those with the response of interest (ventilator dependent or in-hospital deceased) from those without the response of interest (non-ventilator dependent or alive). The optimal cutoff was found by either maximizing the Youden index or minimizing the Euclidean distance of sensitivity and specificity [16]. The predictive ability of the cutoff level was assessed by using the receiver operating characteristic (ROC) curve and calculating the area under the curve (AUC). AUC measures of 0.5 are poor, and measures of 1 are perfect. Odds ratios (ORs) with 95% confidence intervals (CIs) were reported alongside the intraclass correlations (ICCs). Here, ICC is the proportion of total variance in the outcome that is explained by the clustering of similar laboratory results within each patient. To assess differences between levels of gender and comorbidity, the same modeling techniques were repeated by stratifying ferritin and D-dimer cohorts by gender as well as by CCI groups (0, 1–2, 3–4, ≥5).

To assess the generalizability of the results, cross-validations (CVs) were conducted where 20% of patients (and their repeated measurements) from the complete dataset were randomly sampled to be the testing set, and the remaining 80% of patients were retained as the training set. Models were fit on the training set and obtained optimal cutoffs were used to predict outcomes for the testing set. The mean percentage of correct classifications (MPCC) with corresponding standard errors (SE) were reported. Additionally, for validation purposes, optimal cutoff results were tested and reported on a completely independent dataset comprised of patient encounters from July to September 2020. Models applied the same optimal cutoff levels, which were calculated from the previous analysis, to these data. Percentages of correct classification (PCC) were reported. All of these validation analyses were conducted separately for the two predictors (ferritin and D-dimer) and the two outcomes (IVD and in-hospital mortality).

To investigate the difference in lab-level trajectory over time between patients eventually dying and patients remaining alive, mixed-effects exponential regression models were constructed. The predictor was the interaction between elapsed time (time in days since each patient’s first appointment) and a binary indication of death. This provided a lab-level trend over time for those who died as well as for those who remained alive. To account for the inherent differences between each patient, repeated lab measurements were clustered within each patient. Exponentiated coefficients, relating to the percentage change in lab value, with 95% CIs were calculated, along with coefficient of determination (R^2^) values to estimate the percent of variation in ferritin and D-dimer lab levels as explained by the model predictors. Figures were constructed by using model-predicted ferritin and D-dimer levels from a sample of patients. Lab levels were plotted against time for each patient and stratified by death status. 

As a final sensitivity analysis, the optimal cutoff modeling schema was conducted again under two scenarios: (1) without clustering on each patient, and (2) using only the maximum lab result per patient. This was conducted to compare results with that from the full repeated patient lab measurements with the random effect. All statistical tests were two-sided at a significance level of 5% and were conducted using R version 3.6.1 (R Foundation for Statistical Computing).

## 3. Results

### 3.1. Descriptive Statistics

There were 52,411 unique patients infected with COVID-19, and of whom 28.5% (*n* = 14,958) had a valid ferritin lab result and 28.6% (*n* = 15,005) had a valid D-dimer lab result. The median (Q1–Q3) age in years was 61 (49–73) for patients with ferritin results, and 61 (47–73) for patients with D-dimer results (Table 2). Both the ferritin and D-dimer groups had higher percentages of males (ferritin: 53.9% (*n* = 8059); D-dimer: 52.9% (*n* = 7945)). Most patients were from the eastern United States (Table 2). About 17.9% (*n* = 2677) of ferritin patients and 18.3% (*n* = 2744) of D-dimer patients had CCI ≥ 5. Ferritin and D-dimer patients saw, respectively, 25.6% and 24.7% dependent on invasive ventilators, and of those on ventilators, 44.4% and 45.0% died in the hospital (Table 1).

### 3.2. Optimal Cutoffs

**Figure 1:**
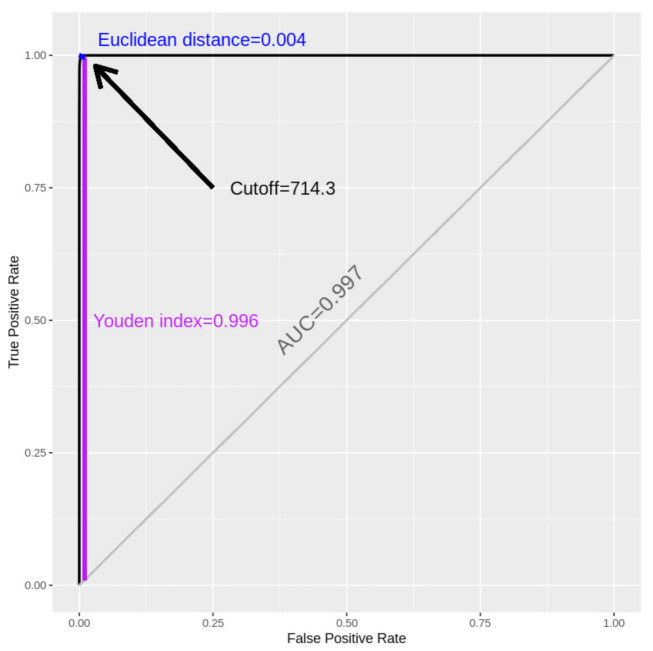
Receiver operating characteristic (ROC) for the optimal cutoff of ferritin (ng/mL) in predicting in-hospital mortality.

Optimal cutoffs of ferritin (714.3 ng/mL) and D-dimer (2.1 mg/L) revealed AUCs ≥ 0.99 for in-hospital mortality. Patients with a ferritin ≥714.3 ng/mL had 3.7 (95% CI: 2.8–4.8) higher odds of in-hospital mortality compared to those with a lower ferritin value (Table 3). Figure 1 provides visualizing on how the optimal cutoff is chosen on the ROC curve. Similarly, optimal cutoffs for ferritin (501.6 ng/mL) and D-dimer (2.0 mg/L) revealed AUCs ≥ 0.99 for IVD. Specifically, patients with a ferritin value of ≥501.6 had 3.4 (95% CI: 2.8–4.2) higher odds of IVD compared to those below this cutoff, while patients with D-dimer ≥ 2.0 had 6.4 (95% CI: 5.1–8.1) higher odds of IVD compared to those below. Optimal cutoffs for in-house mortality, among females, were lower in serum ferritin (433.3 ng/mL) and D-dimer (1.9 mg/L) compared to males (740.0 ng/mL and 2.5 mg/L, respectively). Table 3 also shows that optimal cutoffs for IVD, among females, were lower in ferritin (270.0 ng/mL) and D-dimer (1.3 mg/L) compared to males (860.4 ng/mL and 2.3 mg/L, respectively). 

There were also differences across the CCI. For ferritin, the optimal cutoff started low at index “0”, increased across indices “1–2” and “3–4” and then decreased in index “≥5”, for both in-hospital mortality and IVD. For D-dimer, patients with a CCI of “0” had the highest cutoff level (6.5) for in-hospital mortality, but this cutoff decreased for the other comorbidity indices. Patients with a CCI of “0” had the lowest D-dimer cutoff for IVD (1.4), and the cutoff fluctuated over the other indices. ICCs were reported for all models, all of which were above 0.94, indicating high correlations of lab results among the same subjects (Table 3).

### 3.3. Validation

Firstly, CV was conducted over 100 iterations, each time providing the PCC to be able to distinguish outcomes from non-outcomes, and the average PCC (MPCCs with SEs) were reported in Table 4. All models, under all scenarios, reported excellent classification ability with PCCs and MPCCs all reporting 0.996 and higher, with very little variation. Secondly, the results of testing the optimal cutoffs on the independent September data refresh, including data from July until September 2020, also indicated excellent performance with all PCCs of 0.999 (rightmost column of Table 4).

### 3.4. Trajectories of Measurements

**Figure 2:**
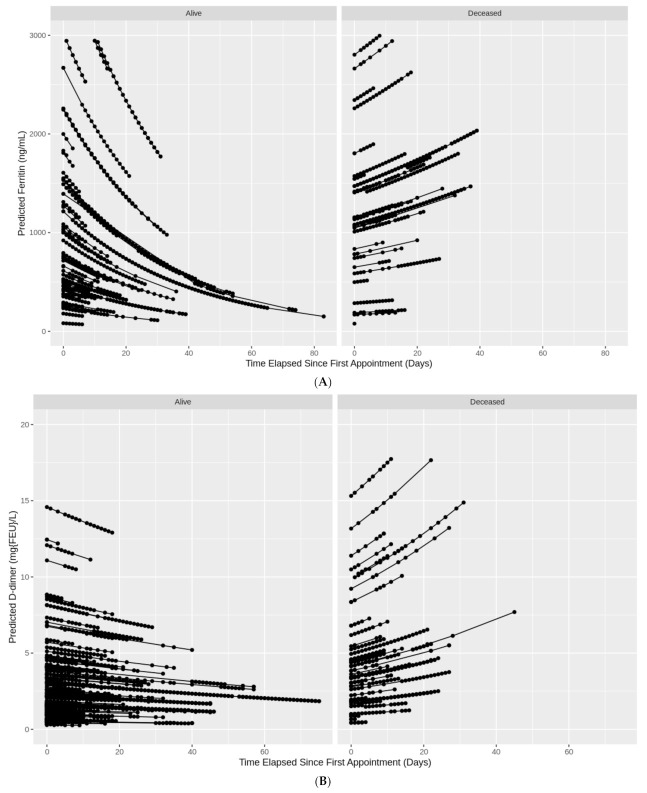
(**A**) Individual trajectories of ferritin levels over time in COVID-19 patients by death status. (**B**) Individual trajectories of D-dimer levels over time in COVID-19 patients by death status.

Table 5 reports the percentage change in ferritin and D-dimer lab levels across time (10-day increments) for those who died and those who remained alive, while clustering repeated lab measurements on each patient. Ferritin significantly decreased over time for those who remained alive ($e^{\hat{\beta }}$ (95% CI): 0.85 (0.84, 0.86)), yet significantly increased over time for those who eventually died ($e^{\hat{\beta }}$ (95% CI): 1.04 (1.03, 1.05)). D-dimer also significantly decreased over time for those alive ($e^{\hat{\beta }}$ (95% CI): 0.93 (0.92, 0.94)) and significantly increased over time for those who died ($e^{\hat{\beta }}$ (95% CI): 1.14 (1.13, 1.16)). The models predicted 88% of the variation in ferritin and 74% in D-dimer levels. Figure 2A,B illustrate the trends seen in Table 5. Both ferritin and D-dimer levels consistently decreased over time for patients who remained alive, while increasing over time for patients who eventually died.

### 3.5. Sensitivity Analysis

Sensitivity analyses in Supplemental Tables S4 and S5 show the optimal cutoffs determined in the absence of clustering on each patient, or only using the maximum lab value per patient. Both scenarios show comparable cutoffs; however, the classification ability of all models was extremely reduced, with AUCs ranging from 0.59 to 0.74. 

## 4. Discussion

Due to limited hospital resources, it is critical to understand threshold patterns of serum biomarkers that can predict CSS, which would aid in judicious allocation of resources, bridging the time for the development of an effective vaccine and medical treatment. Here, we identified optimal cutoffs of serum ferritin and D-dimer levels in COVID-19 patients that predict in-hospital mortality and IVD. These cutoffs, for some CCI categories, seem to be comparable to values obtained from initial retrospective studies in Wuhan, China. In a study of 21 patients, Chen et al. found 11 patients with severe COVID-19 infection to have a serum ferritin higher than 800 µg/L [17]. In another retrospective study of 191 patients at the Jinyintan Hospital and Wuhan Pulmonary Hospital, a serum level of >300 µg/L was present in 96% of non-survivors [18]. 

In this study, female patients with COVID-19 have an overall lower optimal serum ferritin and D-dimer cutoff for in-hospital mortality and IVD compared to males. The ratio between genders was almost 1:3 (or 270.0: 860.4) for IVD, which is higher than that among healthy adults, where females have a lower serum ferritin range (12–150 ng/mL) compared to males (12–300 ng/mL) [19]. When examining the trajectory of repeated ferritin and D-dimer levels on subsequent days of illness, we found persistently elevated, time-spaced repeated measurements of ferritin and D-dimer to be highly predictive of patients developing CSS, with imminent need of ventilation, and eventual in-hospital death. We uncovered two distinct trajectory patterns, each representing mortality or survival. The first trajectory pattern consists of rapid elevation, in which repeated serum levels of ferritin and D-dimer continue to increase over time, a pattern that highly correlates with mortality. The second trajectory pattern witnesses decreasing or unchanged subsequent levels of ferritin and D-dimer, which highly correlates with survival. Among patients with low initial ferritin and D-dimer values, but increasing subsequent time-spaced repeated values, there is a high chance of developing CSS, needing invasive ventilation, and experiencing in-hospital mortality. However, patients with high initial ferritin and D-dimer levels, and stable or decreasing subsequent time-spaced repeated values, have a low probability for developing CSS, needing ventilation, and experiencing death. This might indicate distinct biological and physiological feedback mechanisms during SARS-CoV-2 infections among certain susceptible patient groups, since genetic susceptibility factors could contribute to CSS development [20]. 

Among COVID-19 patients, older age and comorbidities such as diabetes or hypertension are predictors of increased COVID-19 related morbidity and mortality [21]. We hypothesized that COVID-19 patients without any comorbidities would possess high serum ferritin and D-dimer levels because they have adequate cardiac and pulmonary reserves and would require a strong COVID-19 related CSS to develop respiratory distress resulting in ventilation and in-hospital mortality. Conversely, in patients with increasing comorbidities, we predicted that a modest increase in serum ferritin and D-dimer due to COVID-19 would result in multiorgan failure, owing to pre-existing limited cardiopulmonary and immune reserves. However, we did not find a clear pattern for optimal cutoff in relation to CCI. As hypothesized, serum ferritin and D-dimer should have the highest optimal cutoff in patients with no comorbidities. However, in our study using CCI, patients with no comorbidities had the lowest serum ferritin and D-dimer optimal cutoff for IVD, and the lowest serum ferritin cutoff for in-house mortality. This finding may represent additional unknown factors (e.g., male sex, blood type A, and other genetic and exposure susceptibility factors, including the triggering of a previous viral infection), or immune responses toward CSS that result in mortality and IVD in relation to COVID-19 infection [20,22]. Furthermore, individuals with no comorbidities likely activate thrombotic responses early after SARS-CoV-2 infection [23], which demonstrates the importance of D-dimer tests being conducted early after admission, especially among younger, generally healthy patients.

Another important finding of our study is that optimal ferritin and D-dimer cutoffs alone are not sufficient information, but must be paired with knowledge of the trajectory of repeated ferritin and D-dimer levels drawn on subsequent days of illness for COVID-19 patients. ICCs indicated exceptionally high correlation of lab values within each patient (ICC ≈ 0.96), and either ignoring this aspect or collapsing down to one value per patient would eliminate this valuable information. The information lost is demonstrated by the stark difference in AUCs between Table 3 (AUC ≈ 0.99) and Supplemental Tables S4 and S5 (AUC ≈ 0.60–0.75). The increase in AUC when considering the entire trajectory of values could partly be due to the increase in the number of observations [15]. Our findings in Supplemental Tables S4 and S5 are supported by a recent study published in August 2020 on 942 patients from a large New York City health system [24]. Using a single measurement, the authors obtained poor to fair accuracy (AUC ≈ 0.60–0.75) for ferritin as a biomarker when they collapsed repeated measurements down. We demonstrated in our study, using logistic random-effects modeling, that considering the entire trajectory of measurements optimizes the accuracy of ferritin as a biomarker (see Table 3 versus Supplemental Tables S4 and S5). Using such methodology, we assumed that sensitivity and specificity were centered around a common mean across the patients (clusters) with some level of variability. Further details on using logistic random-effects models to adjust for correlations between subjects’ measurements when calculating sensitivity and specificity have been shown elsewhere [15]. 

Our optimal cutoffs for ferritin (714.3 ng/mL for in-hospital mortality with AUC = 0.997; and 501.6 for IVD with AUC = 0.996), and D-dimer (2.1 ng/mL for in-hospital mortality with AUC = 0.997; and 2.0 for IVD with AUC = 0.998) were validated by using CV on a testing set, and by using an independent large cohort (Table 4), demonstrating substantial performance.

### 4.1. Limitations

One of the main limitations of studying serum ferritin and D-dimer is working with non-standardized data from blood samples, which lack uniform measurement units due to the variability of various lab tests (see Supplemental Table S1). Furthermore, patients entered this cohort at different timepoints, when experimental treatments and guidelines for COVID-19 care had been changing rapidly, which might have induced some residual confounding. 

### 4.2. Recommendations

In line with the identification protocol of acute coronary syndrome, which uses time-spaced troponin I levels, we propose a similar approach to classify COVID-19 infection. We recommend the development of a standardized protocol with identification of appropriate time gap between repeated values for ferritin and D-dimer in COVID-19 patients. We hypothesize that at least three repeated values of ferritin and D-dimer, spaced over 8 to 24 h, will help identify the appropriate trajectory pattern, similar to serum troponin collection during acute coronary syndrome. The ferritin and D-dimer pneumogram should consider the start point of COVID-19 infection as the development of a fever.

## 5. Conclusions

With many countries experiencing a second or possible third wave of COVID-19, appropriate allocation of health care resources is pivotal in avoiding overcrowding of hospitals and minimizing burden on health care systems. Knowledge and application of ferritin and D-dimer trajectory patterns, in addition to optimal cutoffs, will help allocate valuable resources and make better predictions about disease progression. Time-spaced repeated measurements of serum ferritin and D-dimer will identify patients with high risk of developing CSS who will require intensive care resources. Whether at home, in primary care centers, or temporary COVID-19 hospitals, cytokine trajectory-based care can be performed safely without transfer to a tertiary care center for patients with stable or decreasing serum ferritin and D-dimer trajectories. Ultimately, serum ferritin and D-dimer trajectory plots will provide improved confidence to providers working in remote areas and temporary COVID-19 hospitals in predicting transfer of COVID-19 patients to tertiary care hospitals. 

## Figures and Tables

**Table 1:** Clinical characteristics among COVID-19 patients with valid ferritin and D-dimer laboratory measurements.

Characteristic	Total *n* (% ^1^)	Ferritin *n* (% ^1^)	D-Dimer *n* (% ^1^)
**Total ^4^**	**52,411 (100.00)**	**14,958 (28.5)**	**15,005 (28.6)**
**Charlson weighted comorbidity index**			
0	24,713 (47.2)	4801 (32.1)	5033 (33.5)
1–2	15,065 (28.7)	5413 (36.2)	5170 (34.5)
3–4	5112 (9.8)	2067 (13.8)	2058 (13.7)
≥5	7521 (14.1)	2677 (17.9)	2744 (18.3)
**Chronic diseases ^2^**			
Myocardial infarction	2624 (5.0)	1015 (6.8)	1121 (7.5)
Congestive heart failure	6333 (12.1)	2292 (15.3)	2351 (15.7)
Peripheral vascular disease	4019 (7.7)	1325 (8.9)	1335 (8.9)
Cerebrovascular disease	3999 (7.6)	1542 (10.3)	1577 (10.5)
Dementia	4303 (8.2)	1760 (11.8)	1788 (11.9)
Chronic pulmonary disease	10,815 (20.6)	3205 (21.4)	3162 (21.1)
Rheumatic disease	1112 (2.1)	351 (2.3)	340 (2.3)
Peptic ulcer disease	863 (1.6)	208 (1.4)	243 (1.6)
Mild liver disease	3368 (6.4)	1021 (6.8)	1012 (6.7)
Diabetes without chronic complication	13,606 (26.0)	5646 (37.7)	5442 (36.3)
Diabetes with chronic complication	4152 (7.9)	1531 (10.2)	1623 (10.8)
Hemiplegia or paraplegia	1144 (2.2)	417 (2.8)	445 (3.0)
Renal disease	9913 (18.9)	3798 (25.4)	3963 (26.4)
Any malignancy, including lymphoma and leukemia, except malignant neoplasm of the skin	2878 (5.5)	880 (5.9)	958 (6.4)
Moderate or severe liver disease	550 (1.0)	174 (1.2)	180 (1.2)
Metastatic solid tumor	667 (1.3)	194 (1.3)	185 (1.2)
HIV/AIDS	377 (0.7)	136 (0.9)	127 (0.8)
**Clinical complications**			
Hospitalized	27,774 (53.0)	13,366 (89.4)	12,864 (85.7)
Invasive ventilator dependence (IVD)	6150 (11.7)	3828 (25.6)	3713 (24.7)
In-hospital mortality among ventilator dependent	2665 (43.3 ^3^)	1701 (44.4 ^3^)	1671 (45.0 ^3^)
In-hospital mortality	4502 (8.6)	2480 (16.6)	2523 (16.8)
Ventilator dependence among in-hospital deceased	2665 (59.2 ^3^)	1701 (68.6 ^3^)	1671 (66.2 ^3^)

^1^*n* (column %’s); ^2^ Identified by ICD-10 codes and extend as far back as 1 January 2015 for patients (these are the diseases that make up the Charlson comorbidity index); ^3^ Percentages out of described denominator; ^4^ *n* (row %’s).

**Table 2:** Demographic characteristics among COVID-19 patients with valid ferritin and D-dimer laboratory measurements.

Characteristic	Total *n* (% ^5^)	Ferritin ^1^ *n* (% ^5^)	D-Dimer ^2^ *n* (% ^5^)
**Total ^3^**	**52,411 (100.00)**	**14,958 (28.5)**	**15,005 (28.6)**
**Median Age** (Years) ^4^	53 (35–68)	61 (49–73)	61 (47–73)
**Gender**			
Female	26,512 (50.6)	6862 (45.9)	7021 (46.8)
Male	25,800 (49.2)	8059 (53.9)	7945 (52.9)
Other/Unknown	99 (0.2)	37 (0.2)	39 (0.3)
**Race/Ethnicity**			
Non-Hispanic American Indian or Alaska Native	1070 (2.0)	384 (2.6)	386 (2.6)
Non-Hispanic Asian or Pacific Islander	1447 (2.8)	427 (2.9)	443 (3.0)
Non-Hispanic Black or African American	10,667 (20.4)	3723 (24.9)	3945 (26.3)
Non-Hispanic White	15,048 (28.7)	4396 (29.4)	4744 (31.6)
Non-Hispanic Multiracial/Other/Unknown	5754 (11.0)	2092 (14.0)	2271 (15.1)
Hispanic or Latino	18,425 (35.2)	3936 (26.3)	3216 (21.4)
**Health insurance**			
Private	18,015 (34.4)	4640 (31.0)	4515 (30.1)
Government/Miscellaneous	1853 (3.5)	509 (3.4)	583 (3.9)
Medicaid	8597 (16.4)	2103 (14.1)	2206 (14.7)
Medicare	11,791 (22.5)	4927 (32.9)	5061 (33.7)
Self-Pay	4906 (9.4)	784 (5.2)	757 (5.0)
Missing	7249 (13.8)	1995 (13.3)	1883 (12.5)
**Region of admission ^6^**			
0	6210 (11.8)	2618 (17.5)	2926 (19.5)
1	5593 (10.7)	2213 (14.8)	2476 (16.5)
2	8139 (15.5)	3067 (20.5)	3391 (22.6)
3	9867 (18.8)	2040 (13.6)	1389 (9.3)
4	2701 (5.2)	1092 (7.3)	1255 (8.4)
5	337 (0.6)	81 (0.5)	101 (0.7)
6	1551 (3.0)	382 (2.6)	455 (3.0)
7	3116 (5.9)	806 (5.4)	463 (3.1)
8	3321 (6.3)	881 (5.9)	965 (6.4)
9	9012 (17.2)	1691 (11.3)	1526 (10.2)
Missing	2564 (4.9)	87 (0.6)	58 (0.4)

^1^ Has a valid ferritin lab from a COVID-19 encounter; ^2^ has a valid D-dimer lab from a COVID-19 encounter; ^3^ Unique patients, % is row percentage out of total COVID-19 patients (*n* = 52,411); ^4^ Median (Q1–Q3); ^5^ *n* (column %’s); ^6^ 0 (Connecticut, Massachusetts, Maine, New Hampshire, New Jersey, Rhode Island, Vermont), 1 (Delaware, New York, Pennsylvania), 2 (DC, Maryland, North Carolina, South Carolina, Virginia, West Virginia), 3 (Alabama, Florida, Georgia, Mississippi, Tennessee), 4 (Indiana, Kentucky, Michigan, Ohio), 5 (Iowa, Minnesota, Montana, North Dakota, South Dakota, Wisconsin), 6 (Illinois, Kansas, Missouri, Nebraska), 7 (Arkansas, Louisiana, Oklahoma, Texas), 8 (Arizona, Colorado, Idaho, New Mexico, Nevada, Utah, Wyoming), 9 (Alaska, California, Hawaii, Oregon, Washington).

**Table 3:** Biomarker optimal cut-offs for complications in COVID-19 patients, overall and stratified by gender and Charlson weighted comorbidity index (CCI).

Category		In-Hospital Mortality					InvasiveVentilator Dependence (IVD)				
	**Median (IQR)**	**Cutoff ^1^**	**OR ^4^ (95% CI)**	**AUC**	***n*^5^**	**ICC ^6^**	**Cutoff**	**OR ^4^ (95% CI)**	**AUC**	***n*^5^**	**ICC ^6^**
**Ferritin**	**654 (325–1153) ^2^**	**714.3**	**3.7 (2.8, 4.8)**	**0.997**	**74,758**	**0.956**	**501.6**	**3.4 (2.8, 4.2)**	**0.996**	**74,758**	**0.955**
**Gender**											
Female	413 (202–787)	433.3	5.1 (3.2, 8.1)	0.996	29,981	0.951	270.0	3.0 (2.0, 4.4)	0.998	29,981	0.961
Male	845 (473–1458)	740.0	3.4 (2.4, 4.8)	0.998	44,597	0.961	860.4	3.4 (2.5, 4.6)	0.998	44,597	0.967
**Charlson Index**											
0	653 (323–1180)	610.0	3.8 (1.9, 7.8)	0.994	21,252	0.952	462.2	3.4 (2.1, 5.7)	0.999	21,252	0.961
1–2	641 (334–1148)	1039.5	3.7 (2.2, 6.2)	0.998	28,235	0.959	906.9	4.0 (2.6, 6.4)	0.998	28,235	0.966
3–4	625 (317–1270)	1613.9	8.3 (3.5, 19.4)	0.998	11,799	0.963	1194.7	2.4 (1.1, 5.0)	0.997	11,799	0.968
≥5	710 (318–1456)	786.4	3.5 (2.1, 5.7)	0.996	13,472	0.955	677.9	3.3 (2.0, 5.3)	0.995	13,472	0.962
**D-dimer**	**1.7 (0.8–4.0)**	**2.1 ^3^**	**6.8 (5.3, 8.8)**	**0.997**	**79,643**	**0.953**	**2.0**	**6.4 (5.1, 8.1)**	**0.998**	**79,643**	**0.958**
**Gender**											
Female	1.5 (0.8–3.6)	1.9	5.9 (4.0, 8.7)	0.996	32,121	0.948	1.3	5.4 (3.8, 7.7)	0.998	32,121	0.955
Male	1.8 (0.9–4.3)	2.5	7.0 (5.0, 9.9)	0.997	47,387	0.957	2.3	7.9 (5.7, 11.0)	0.999	47,387	0.961
**Charlson Index**											
0	1.3 (0.7–3.3)	6.5	7.1 (3.3, 15.0)	0.997	22,078	0.950	1.4	5.2 (3.4, 8.0)	0.999	22,078	0.950
1–2	1.7 (0.8–3.9)	1.4	8.8 (5.3, 14.7)	0.998	28,975	0.957	1.8	10.5 (6.9, 16.1)	0.999	28,975	0.959
3–4	1.8 (0.9–4.5)	2.6	5.5 (2.9, 10.6)	0.999	12,654	0.961	2.6	6.2 (3.2, 11.9)	0.997	12,654	0.964
≥5	2.1 (1.1–4.5)	1.3	7.2 (4.2, 12.3)	0.998	15,936	0.961	1.9	4.8 (3.1, 7.5)	0.997	15,936	0.964

^1^ Labs above this value flagged as having outcome, labs below flagged as not having outcome; ^2^ Ferritin: ng/mL; ^3^ D-dimer: mg {FEU}/L; ^4^ Odds of outcome for those with log(lab) greater than or equal to log(cutoff) compared to those below log(cutoff); ^5^ Unique lab indications; ^6^ Intraclass Correlation.

**Table 4:** Cross-validation diagnostics of ferritin and D-dimer optimal cutoffs in predicting in-hospital mortality and invasive ventilator dependence.

Outcome	Predictor	PCC ^1^ for the Complete Set ^4^	MPCC ^2^ (SE ^3^) for the Training Set ^5^	MPCC (SE) for the Test Set ^6^	PCC for the Independent Set ^7^
**In-Hospital Mortality**					
	Ferritin	0.997	0.999 (3.59 × 10^−7^)	0.996 (3.57 × 10^−5^)	0.999
	D-Dimer	0.997	0.999 (4.55 × 10^−7^)	0.997 (3.45 × 10^−5^)	0.999
**Invasive Ventilator Dependence**					
	Ferritin	0.996	0.999 (4.33 × 10^−7^)	0.997 (2.42 × 10^−5^)	0.999
	D-Dimer	0.998	0.999 (4.21 × 10^−7^)	0.998 (2.04 × 10^−5^)	0.999

^1^ Percentage correctly classified; ^2^ Mean percentage correctly classified (100 iterations); ^3^ Standard error; ^4^ Data from January through June 2020; ^5^ 80% of the data from January through June 2020; ^6^ 80% of the data from January through June 2020; ^7^ Data for validation from July until September 2020, sample sizes for: ferritin (encounter, *n* = 98,050; unique patients, *n* = 29,236) D-dimer (encounter, *n* = 128,340; unique patients, *n* = 35,932).

**Table 5:** Associations of elapsed time and death status with ferritin and D-dimer levels.

	Ferritin		D-Dimer	
	$e^{\hat{\beta }}$^2^ (95% CI)	R^2^	$e^{\hat{\beta }}$^2^ (95% CI)	R^2^
**Elapsed time ^1^ by death status**		0.88		0.74
Alive	0.85 (0.84, 0.86)		0.93 (0.92, 0.94)	
Deceased	1.04 (1.03, 1.05)		1.14 (1.13, 1.16)	

^1^ Time (for every 10 days) since first appointment; ^2^ Exponentiated coefficients (mixed-effect exponential regression model clustering on patients) relating to percentage change in expected ferritin/D-dimer level across each one-unit increase of the predictor (interaction between elapsed time and death status).

## Data Availability

The datasets generated and analyzed during the current study are not publicly available due to restrictions by Cerner who owns the data. Data may be accessed by signing a data sharing agreement with Cerner and covering any applicable costs.

## Supplementary Materials

Supplementary materials are available online at https://www.mdpi.com/1999-4915/13/3/419/s1.

## Abbreviations

SARS-CoV-2	novel coronavirus
CSS	cytokine storm syndrome
IVD	invasive ventilator dependence
LOINC	Logical Observation Identifiers Names and Codes
IRB	institutional review board
CCI	Charlson comorbidity index
ROC	receiver operating characteristic
AUC	area under the curve
CI	confidence interval
ICC	intraclass correlation
CV	cross-validation
MPCC	mean percentage of correct classifications
SE	standard error
PCC	percentages of correct classification
R^2^	coefficient of determination

## References

[1] Huang C., Wang Y., Li X., Ren L., Zhao J., Hu Y., Zhang L., Fan G., Xu J., Gu X. (2020). Clinical features of patients infected with 2019 novel coronavirus in Wuhan, China. Lancet.

[2] Mehta P., McAuley D.F., Brown M., Sanchez E., Tattersall R.S., Manson J.J. (2020). COVID-19: Consider cytokine storm syndromes and immunosuppression. Lancet.

[3] Lei J., Li J., Li X., Qi X. (2020). CT imaging of the 2019 novel Coronavirus (2019-nCoV) pneumonia. Radiology.

[4] Kernan K.F., Carcillo J.A. (2017). Hyperferritinemia and inflammation. Int. Immunol..

[5] Eloseily E.M., Weiser P., Crayne C.B., Haines H., Mannion M.L., Stoll M.L., Beukelman T., Atkinson T.P., Cron R.Q. (2020). Benefit of anakinra in treating pediatric secondary hemophagocytic Lymphohistiocytosis. Arthritis Rheumatol..

[6] Colafrancesco S., Alessandri C., Conti F., Priori R. (2020). COVID-19 gone bad: A new character in the spectrum of the hyperferritinemic syndrome?. Autoimmun. Rev..

[7] Taneri P.E., Gómez-Ochoa S.A., Llanaj E., Raguindin P.F., Rojas L.Z., Roa-Díaz Z.M., Salvador D., Groothof D., Minder B., Kopp-Heim D. (2020). Anemia and iron metabolism in COVID-19: A systematic review and meta-analysis. Eur. J. Epidemiol..

[8] Iba T., Levy J.H., Levi M., Connors J.M., Thachil J. (2020). Coagulopathy of Coronavirus disease 2019. Crit. Care Med..

[9] Shorr A.F., Thomas S.J., Alkins S.A., Fitzpatrick T.M., Ling G.S. (2002). D-dimer correlates with proinflammatory cytokine levels and outcomes in critically Ill patients. Chest.

[10] Yu B., Li X., Chen J., Ouyang M., Zhang H., Zhao X., Tang L., Luo Q., Xu M., Yang L. (2020). Evaluation of variation in D-dimer levels among COVID-19 and bacterial pneumonia: A retrospective analysis. J. Thromb. Thrombolysis.

[11] Cerner (2020). COVID-19 De-Identified Data Cohort Access Offer for Academic Researchers. https://www.cerner.com/-/media/covid-19/response/2263471793_covid-19-de-identified-data-cohort-access-offer-faq_v1.aspx.

[12] Charlson M.E., Pompei P., Ales K.L., MacKenzie C. (1987). A new method of classifying prognostic comorbidity in longitudinal studies: Development and validation. J. Chronic Dis..

[13] Quan H., Sundararajan V., Halfon P., Fong A., Burnand B., Luthi J.-C., Saunders L.D., Beck C.A., Feasby T.E., Ghali W.A. (2005). Coding algorithms for defining comorbidities in ICD-9-CM and ICD-10 administrative data. Med Care.

[14] Menendez M.E., Neuhaus V., Van Dijk N.C., Ring D. (2014). The elixhauser comorbidity method outperforms the Charlson index in predicting inpatient death after orthopaedic surgery. Clin. Orthop. Relat. Res..

[15] Genders T.S.S., Spronk S., Stijnen T., Steyerberg E.W., Lesaffre E., Hunink M.G.M. (2012). Methods for calculating sensitivity and specificity of clustered data: A tutorial. Radiology.

[16] Indrayan A., Malhotra R.K. (2017). Medical Biostatistics.

[17] Chen G., Wu D., Guo W., Cao Y., Huang D., Wang H., Wang T., Zhang X., Chen H., Yu H. (2020). Clinical and immunological features of severe and moderate coronavirus disease 2019. J. Clin. Investig..

[18] Zhou F., Yu T., Du R., Fan G., Liu Y., Liu Z., Xiang J., Wang Y., Song B., Gu X. (2020). Clinical course and risk factors for mortality of adult inpatients with COVID-19 in Wuhan, China: A retrospective cohort study. Lancet.

[19] Adams P. (2008). Management of elevated serum ferritin levels. Gastroenterol. Hepatol..

[20] Canna S.W., Behrens E.M. (2012). Making sense of the cytokine storm: A conceptual framework for understanding, diagnosing, and treating hemophagocytic syndromes. Pediatr. Clin. N. Am..

[21] Gandini O., Criniti A., Gagliardi M., Ballesio L., Giglio S., Balena A., Caputi A., Angeloni A., Lubrano C. (2020). Sex-disaggregated data confirm serum ferritin as an independent predictor of disease severity both in male and female COVID-19 patients. J. Infect..

[22] Zietz M., Tatonetti N.P. (2020). Testing the association between blood type and COVID-19 infection, intubation, and death. MedRxiv.

[23] Zhang L., Yan X., Fan Q., Liu H., Liu X., Liu Z., Zhang Z. (2020). D-dimer levels on admission to predict in-hospital mortality in patients with Covid-19. J Thromb Haemost..

[24] Feld J., Tremblay D., Thibaud S., Kessler A., Naymagon L. (2020). Ferritin levels in patients with COVID-19: A poor predictor of mortality and hemophagocytic lymphohistiocytosis. Int. J. Lab. Hematol..

