# Supplementary material for: Prognostic Values of Serum Ferritin and D-Dimer Trajectory in Patients with COVID-19
Source: Viruses. 2021 Mar 5;13(3):419. doi: 10.3390/v13030419 (PMC7998367; doi:10.3390/v13030419)
Supplement: Supplementary file 1 [file viruses-13-00419-s001.pdf]

Supplemental Tables

Supplemental Table S1: List of codes identifying Ferritin and D-dimer

Code Type	Code
**Ferritin**	
LOINC	20567-4, 2276-4, 24373-3
**D-dimer**	
LOINC	15129-0, 15179-5, 29280-5, 30240-6, 3247-4, 42727-8, 48058-2, 48065-7, 48066-5, 48067-3, 71427-9, 7799-0, 91556-1

Supplemental Table S2: List of codes identifying invasive ventilator dependence (IVD)

Code Type	Code
CPT-4 (HCPCS Level I)	31500, 31600, 31601, 31603, 31605, 31610, 31615, 31820, 31825, 31830
ICD-9-CM Diagnoses	519, 519.02, 519.09, V44.0, V46.11, V55.0
ICD-9-CM Procedures	31.1, 31.74, 96.04, 97.23
ICD-10-CM	J95.0, J95.00, J95.01, J95.02, J95.03, J95.04, J95.09, Z43.0, Z93.0, Z99.11
ICD-10-PCS	0B110F4, 0B113F4, 0B21XEZ, 0B21XFZ, 0BH13EZ, 0BH17EZ, 0BH18EZ, 0BP10FZ, 0BP1XFZ, 0BW10FZ, 0BW13FZ, 0BW18FZ, 0BW1XFZ
LOINC	19834-1, 20058-4, 20077-4, 20079-0, 20112-9, 20116-0, 33438-3, LA11274-0
SNOMED CT	2267008, 6774004, 21619009, 55622001, 59006007, 68033004, 82872004, 112798008, 129121000, 161685006, 173070006, 232613003, 232685002, 274969000, 302108003, 302323001, 307007002, 348576000, 385858000, 448621002, 709146000

Supplemental Table S3: List of chronic diseases involved in CCI, with corresponding ICD-10 codes

Chronic Disease	ICD-10 Codes
Myocardial infarction	I21.x, I22.x, I25.2
Congestive heart failure	I09.9, I11.0, I13.0, I13.2, I25.5, I42.0, I42.5 - I42.9, I43.x, I50.x, P29.0
Peripheral vascular disease	I70.x, I71.x, I73.1, I73.8, I73.9, I77.1, I79.0, I79.2, K55.1, K55.8, K55.9, Z95.8, Z95.9
Cerebrovascular disease	G45.x, G46.x, H34.0, I60.x - I69.x
Dementia	F00.x - F03.x, F05.1, G30.x, G31.1
Chronic pulmonary disease	I27.8, I27.9, J40.x - J47.x, J60.x - J67.x, J68.4, J70.1, J70.3
Rheumatic disease	M05.x, M06.x, M31.5, M32.x - M34.x, M35.1, M35.3, M36.0
Peptic ulcer disease	K25.x - K28.x
Mild liver disease	B18.x, K70.0 - K70.3, K70.9, K71.3 - K71.5, K71.7, K73.x, K74.x, K76.0, K76.2 - K76.4, K76.8, K76.9, Z94.4
Diabetes without chronic complication	E10.0, E10.1, E10.6, E10.8, E10.9, E11.0, E11.1, E11.6, E11.8, E11.9, E12.0, E12.1, E12.6, E12.8, E12.9, E13.0, E13.1, E13.6, E13.8, E13.9, E14.0, E14.1, E14.6, E14.8, E14.9
Diabetes with chronic complication	E10.2 - E10.5, E10.7, E11.2 - E11.5, E11.7, E12.2 - E12.5, E12.7, E13.2 - E13.5, E13.7, E14.2 - E14.5, E14.7
Hemiplegia or paraplegia	G04.1, G11.4, G80.1, G80.2, G81.x, G82.x, G83.0 - G83.4, G83.9

Renal disease	I12.0, I13.1, N03.2 - N03.7, N05.2 - N05.7, N18.x, N19.x, N25.0, Z49.0 - Z49.2, Z94.0, Z99.2
Any malignancy, including lymphoma and leukaemia, except malignant neoplasm of skin	C00.x - C26.x, C30.x - C34.x, C37.x - C41.x, C43.x, C45.x - C58.x, C60.x - C76.x, C81.x - C85.x, C88.x, C90.x - C97.x
Moderate or severe liver disease	I85.0, I85.9, I86.4, I98.2, K70.4, K71.1, K72.1, K72.9, K76.5, K76.6, K76.7
Metastatic solid tumour	C77.x - C80.x
AIDS/HIV	B20.x - B22.x, B24.x

Supplemental Table S4: Biomarker optimal cut-offs for complications in COVID-19 patients (no clustering on patient)

Variables	In-Hospital Mortality			Invasive Ventilator Dependence		
	Cutoff[1]	AUC	n[5]	Cutoff[1]	AUC	n[5]
**Ferritin**	657.75[2]	0.60	91,550	528.98[2]	0.59	91,550
**D-dimer**	1.91[3]	0.66	93,611	1.41[3]	0.64	93,611

[1] Optimal cutoff technique
[2] Ferritin: ng/mL
[3] D-dimer: mg {FEU}/L
[4] odds of outcome for those with log(lab) greater than or equal to log(cutoff) compared to those below log(cutoff)
[5] unique lab indications

Supplemental Table S5: Biomarker optimal cut-offs for complications in COVID-19 patients (max lab per patient)

Variables	In-Hospital Mortality			Invasive Ventilator Dependence		
	Cutoff[1]	AUC	n[5]	Cutoff[1]	AUC	n[5]
**Ferritin**	657.07[2]	0.67	21,828	752.28[2]	0.65	21,828
**D-dimer**	2.01[3]	0.74	21,616	2.08[3]	0.71	21,616

[1] Optimal cutoff technique
[2] Ferritin: ng/mL
[3] D-dimer: mg {FEU}/L
[4] odds of outcome for those with log(lab) greater than or equal to log(cutoff) compared to those below log(cutoff)
[5] one unique lab indication per patient, unique patients
